# Mesenchymal stromal/stem cell (MSC)-derived exosomes in clinical trials

**DOI:** 10.1186/s13287-023-03287-7

**Published:** 2023-04-07

**Authors:** Ahmed Lotfy, Noha M. AboQuella, Hongjun Wang

**Affiliations:** 1 Department of Surgery, Medical University of South Carolina, Charleston, SC 29425 USA; 2 International Graduate Program Medical Neuroscience, Charité-Universitätsmedizin Berlin, Berlin, Germany; 3 Ralph H. Johnson Veterans Affairs Medical Center, Charleston, SC USA

**Keywords:** Mesenchymal stromal/stem cell, MSC, Extracellular vesicles, Exosomes, Clinical trials

## Abstract

Mesenchymal stromal/stem cells (MSCs) are widely utilized in cell therapy because of their robust immunomodulatory and regenerative properties. Their paracrine activity is one of the most important features that contribute to their efficacy. Recently, it has been demonstrated that the production of various factors via extracellular vesicles, especially exosomes, governs the principal efficacy of MSCs after infusion in experimental models. Compared to MSCs themselves, MSC-derived exosomes (MSC-Exos) have provided significant advantages by efficiently decreasing unfavorable adverse effects, such as infusion-related toxicities. MSC-Exos is becoming a promising cell-free therapeutic tool and an increasing number of clinical studies started to assess the therapeutic effect of MSC-Exos in different diseases. In this review, we summarized the ongoing and completed clinical studies using MSC-Exos for immunomodulation, regenerative medicine, gene delivery, and beyond. Additionally, we summarized MSC-Exos production methods utilized in these studies with an emphasis on MSCs source, MSC-Exos isolation methods, characterization, dosage, and route of administration. Lastly, we discussed the current challenges and future directions of exosome utilization in different clinical studies as a novel therapeutic strategy.

## Introduction

Mesenchymal stromal/stem cells (MSCs) are one of the most frequently used stem cells in cellular therapy trials based on their immunomodulation, tissue regeneration, and protective functions [1–3]. Previously, exogenous-infused MSCs were thought to exert their protective effects by migrating to damaged tissues, engrafting, and interacting with other cells after infusion. However, several recent preclinical studies and clinical trials have reported that the therapeutic effect of MSCs is exerted through the paracrine production of growth factors, chemokines, and cytokines [4–6]. Nonetheless, the mechanism of action of MSCs is still not completely understood and requires further investigation. Despite their high curative efficacy, MSC therapy has some drawbacks, such as difficulty in generating a consistent source of cells with a stable phenotype, infusion-related toxicities resulting from the cells physically trapped in the lung microvasculature, and others [7–10].

MSCs produce multiple extracellular vesicles (EVs): exosomes (30–150 nm in diameter), microvesicles (150–500 nm in diameter), and apoptotic bodies (800–500 nm in diameter). EVs are thought to serve as paracrine mediators between MSCs and their target cells [11, 12]. In addition, MSC-derived exosomes (MSC-Exos) can recapitulate the biological potential of MSCs; therefore, they may substitute for cell therapy to achieve cell-free therapy [13, 14]. The advantages of exosome utilization, when compared to their cellular counterparts, include a higher safety profile mainly due to their nanoscale size. In contrast to MSCs, which have a diameter of 30–60 μm, nanosized exosomes can transfer efficiently to specific tissues after administration without aggregation in the lung microvasculature [15, 16], avoiding the possibility of a pulmonary embolism caused by administrated cells [17]. Moreover, MSC-Exos could be isolated from immortalized MSCs and used for cell therapy, which is not applicable to the immortalized cells themselves.

MSC-Exos engage in intercellular communication, serving as carriers to deliver proteins, mRNA, and microRNAs into targeted cells [11]. More than 304 proteins and 150 microRNAs have been found in MSC-Exos in addition to other bioactive molecules [18–20]. All these bioactive molecules show a promising therapeutic effect on tissue recovery by the maintenance and recruitment of their endogenous stem cells, inhibition of apoptosis, immunomodulation, and stimulation of angiogenesis [21]. Some studies have proposed that the phenotype and function of MSC-Exos may vary depending on the source of MSCs [15]. According to comparative RNA sequencing studies of the MSC-Exos from human bone marrow and adipose tissues, there are differences identified in tRNA species that are defined by Sox2, POU5F1A/B, and Nanog gene expression, likely related to the differentiation status of MSCs [22]. Furthermore, the sources of MSCs are thought to affect the therapeutic effects of MSC-Exos [15]. A comparative study has demonstrated that the in vivo biological and therapeutic impacts of MSC-Exos from different human tissue sources, such as the endometrium, bone marrow, and adipose tissues, are different [23]. These results confirm that the inherent variations of MSC-Exos due to their various sources can affect their therapeutic efficacy.

Besides their intrinsic properties, MSC-Exos are exemplary vehicles to deliver different molecules to targeted cells, including curative genes, drugs, enzymes, and RNA [13]. Numerous studies have demonstrated that MSC-Exos can protect molecules against disintegration and facilitate their cellular absorption through endocytosis [13]. Moreover, MSC-Exos may be an ideal carrier system to transiently modify specific processes in target cells [24] due to its ability to alter their exosomal surface to promote cell-type-specific targeting [25]. These features make MSC-Exos a promising tool for cell-free therapy for various debilitating disorders.

## Therapeutic potential of MSC-Exos in preclinical studies

MSC-Exos are a promising therapeutic product because they can carry various molecules and protect their cargo from degradation in the blood [26]. There is ample evidence from preclinical studies indicating that MSC-Exos can prevent or treat different diseases in animal models [27]. Additionally, MSC-Exos have demonstrated their effectiveness in animal models of neuro-related disorders, such as epilepsy [28], Parkinson’s disease [29], and stroke [30]. Moreover, MSC-Exos can exert immunomodulatory effects in animal models of autoimmune diseases, such as multiple sclerosis [31], rheumatoid arthritis [32], and type 1 diabetes [33]. Furthermore, MSC-Exos are effective in cardiac, hepatic, and renal regeneration [14, 34, 35]. In summary, the demonstrated efficacy of MSC-Exos in animal disease models suggests that MSC-Exos may provide a promising therapeutic approach for a variety of diseases in human.

The biological function of MSC-Exos from different sources shares most properties; however, they may also vary in functionality [36, 37]. For instance, MSC-Exos extracted from adipose tissues have a better angiogenic capability than those extracted from the bone marrow (BM) [38]. However, BM-MSCs-derived exosomes (BM-MSC-Exos) can inhibit IFN-γ secreted by T cells and can have an immunomodulatory effect as well as an anti-inflammatory effect [36]. In preclinical studies, the specific minimal effective dose of MSC-Exos has not been determined; though in mouse models, 10–100 μg of MSC-Exos has been used [39]. Interestingly, Maria and colleagues showed that the dose with the highest therapeutic efficacy was not necessarily the highest dose attempted [40]. Another important factor affecting the therapeutic effect of MSC-Exos is the route of delivery. Different routes of administration have been evaluated; Although the most commonly used route in preclinical studies is intravenous (IV) injection [41], intraperitoneal and subcutaneous injection of MSC-Exos led to more accumulation in organs such as the pancreas [42]. Therefore, the impacts of several factors, such as the source of MSC-Exos, minimal effective dose, and route of delivery, on their efficacy, need to be investigated further in preclinical studies in a disease-specific manner.

## MSC-Exos in clinical applications

**Fig. 1:**
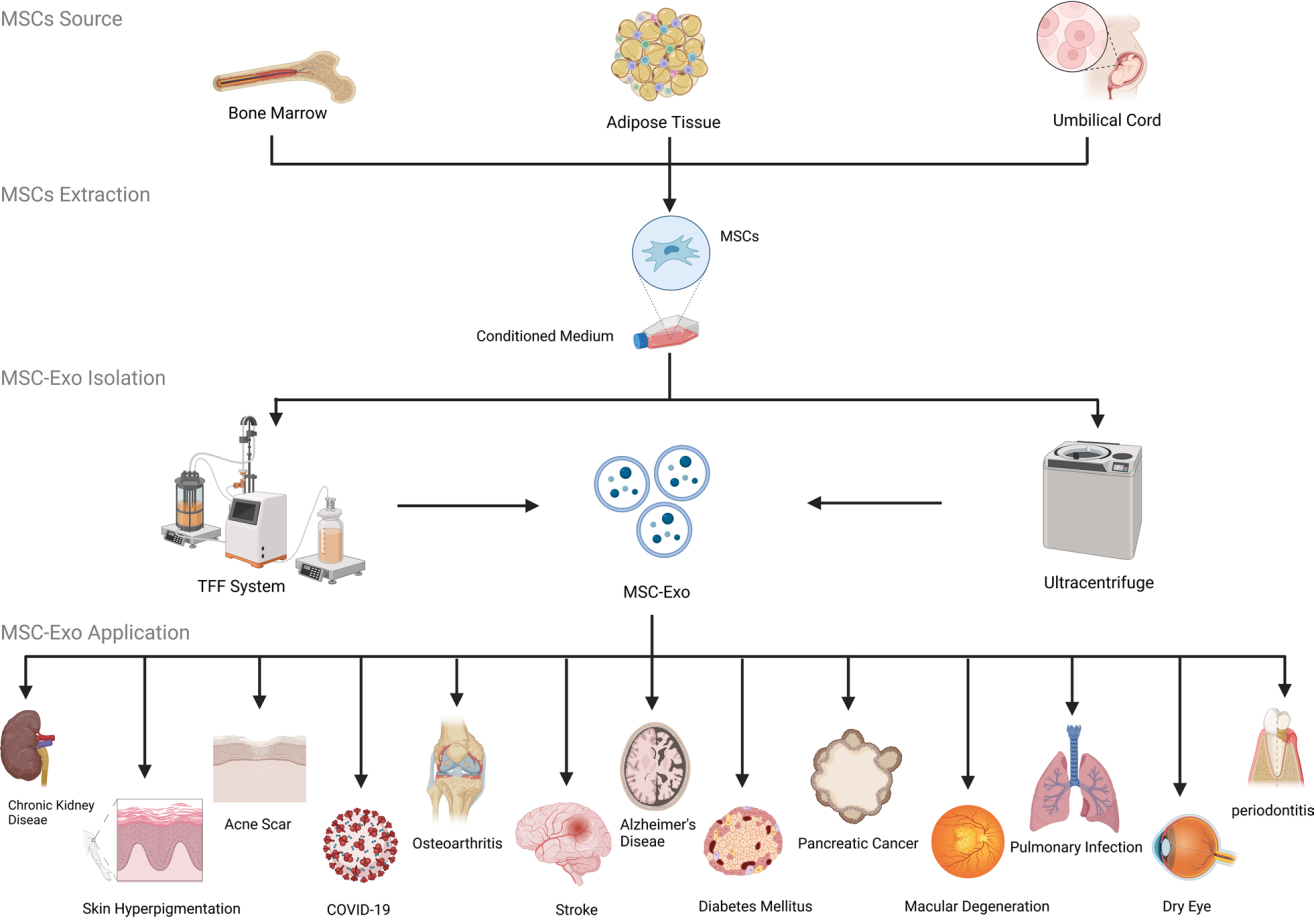
Summary of the MSC-Exos cell sources, isolation approaches and utilization in different diseases

Seven published clinical studies (Table 1) and 14 ongoing clinical trials (Table 2) (as of September 2022) tested MSC-Exos as a therapeutic agent against different diseases, including acute respiratory distress syndrome (ARDS), kidney diseases, graft-versus-host disease (GvHD), osteoarthritis, stroke, Alzheimer’s disease, and type 1 diabetes (Fig. 1). We summarized these studies with an emphasis on the sources, doses, administration routes, characterizations, isolation methods, and potential mechanism of action of MSC-Exos in each study.

**Table 1:** Published MSC-Exos clinical studies

No	Clinical trial ID	Study title	Phase	Number of patients	Disease	Study type	Source of MSC	Source (allogeneic –autologous)	Route of administration	Dose	Treatment outcome (Exos vs control except in open-labeled study indicated by *)	Refs
1	N/A	Umbilical cord mesenchymal stem cells derived extracellular vesicles can safely ameliorate the progression of chronic kidney diseases	2/3	40	Chronic kidney disease	Single-center, randomized, placebo-controlled, phase II/III clinical pilot study	Umbilical cord blood	Allogeneic	IV/IA	Two doses (one week apart) of exosomes 100 μg/kg/dose	Significant improvement in eGFR, serum creatinine level, blood urea, and UACR during the 12-month study period	(52)
2	N/A	Skin Brightening Efficacy of Exosomes Derived from Human Adipose Tissue-Derived Stem/Stromal Cells: A Prospective, Split-Face, Randomized Placebo-Controlled Study	N/A	21	Skin hyperpigmentation	A prospective, split-face, randomized placebo-controlled study	Adipose tissue	Allogeneic	Local	0.2 g of MSC-Exos twice a day for 8 weeks	Significantly reduced the amount of melanin for 2 months	(55)
3	N/A	Combination Treatment with Human Adipose Tissue Stem Cell-derived Exosomes and Fractional CO_2_ Laser for Acne Scars: A 12-week Prospective, Double-blind, Randomized, Split-face Study	N/A	25	Acne scars	N/A	Adipose tissue	Allogeneic	Local	MSC-Exos gel (9.78 × 10^10^ particles/ml)	Reduced the size of skin pores and skin surface scabrously from baseline on the treated side	(58)
4	N/A	MSC-Exosomes and GvHD	N/A	1	GvHD	Case report	Bone marrow	Allogeneic	IV	Four-time dosage (2–3 days apart of each dose) of exosomes produced from 4 × 10^7^ MSCs	The clinical GvHD symptoms improved significantly shortly after MSC-Exos therapy. The cutaneous and mucosal GvHD showed a notable restrain within 2 weeks, which was stable even after 4 months	(59)
5	NCT04657458	BM-MSC-Exos and COVID-19	N/A	24	COVID-19	Prospective non-randomized open-label cohort study	Bone marrow (ExoFlo)	Allogeneic	IV	A single dose of exosomes produced from 1–10 × 10^6^ MSCs/kg	* Patient survival rate was 83%; about 71% of the patients were cured, 13% remained in critical but stable condition, and 16% died for reasons unrelated to the treatment	(61)
6	N/A	IRB Approved Pilot Safety Study of an Extracellular Vesicle Isolate Product Evaluating the Treatment of Osteoarthritis in Combat-Related Injuries	N/A	33	Osteoarthritis	Pilot study	Bone marrow (ExoFlo)	Allogeneic	Local	A single dose of ExoFlo (exosomes produced from 1–10 × 10^6^ MSCs/kg)	After six-month follow-up, the average patient improved 77% in BPI, 80% in ODI, 76% in LEFS, 51% in UEFS, and 77% in QD	(62)
7	NCT04313647	A Tolerance Clinical Study on Aerosol Inhalation of Mesenchymal Stem Cells Exosomes In Healthy Volunteers	½	24	Healthy	Non-randomized/open label	Adipose tissue	Allogeneic	Inhalation	2 × 10^8^ to 16 × 10^8^ particles	MSC-Exos were safe; the volunteers tolerated the infusion well and did not show adverse reactions within the week after nebulization	(63)

*Abbreviations* MSCs: Mesenchymal stromal/stem cells; BPI: Brief Pain Inventory; ODI: Oswestry Disability Index; LEFS: Lower Extremity Functional Scale; UEFS: Upper Extremity Functional Scale; QD: QuickDASH

**Table 2:** Ongoing MSC-Exos clinical studies

No	Clinical trial ID	Study title	Phase	Number of patients	Disease	Study type	Source of MSC	Source (allogeneic–autologous)	Route of administration	Dose
1	NCT02138331	Effect of Microvesicles and Exosomes Therapy on β-cell Mass in Type I Diabetes Mellitus (T1D)	2/3	N/A	Type I diabetes mellitus	Interventional open-label	Umbilical cord blood	Allogeneic	IV	Two doses (one week apart) of exosomes produced from 1.22–1.51 × 10^6^ MSCs/kg
2	NCT03437759	MSC-Exos Promote Healing of MHs	1	N/A	Macular degeneration	Randomized	Umbilical cord tissue	Allogeneic	Local	50 μg or 20 μg MSC-Exos
3	NCT03384433	Allogeneic Mesenchymal Stem Cell-Derived Exosome in Patients with Acute Ischemic Stroke	1/2	5	Ischemic stroke	Randomized, single-blinded, placebo-controlled	Bone marrow	Allogeneic	Stereotaxis/intraparenchymal	Single dose of 200 mg MSC-Exos
4	NCT04276987	A Pilot Clinical Study on Inhalation of Mesenchymal Stem Cells Exosomes Treating Severe Novel Coronavirus Pneumonia	1	24	COVID-19	Pilot/open-Label	Adipose tissue	Allogeneic	Aerosol inhalation	5 times of MSC-derived exosomes (MSC-Exos) (2 × 10^8^ nanovesicles/3 ml at Day 1, Day 2, Day 3, Day 4, Day 5)
5	NCT04491240	SARS-CoV-2 Associated Pneumonia	1	30	SARS-CoV-2	Interventional	N/A	Allogeneic	Inhalation	Twice a day during 10 days inhalation of 3 ml special solution contained 0.5–2 × 10^10^ of nanoparticles
6	NCT04356300	Multiple Organ Dysfunction Syndrome After Surgical Repair of Acute Type A Aortic Dissection	N/A	60	Multiple organ dysfunction syndrome	Interventional	N/A	Autologous	IV	150 mg once a day for 14 times
7	NCT04213248	Effect of UC-MSC-Exos on Dry Eye in Patients With cGVHD	1/2	27	Dry eye	Single group assignment	Umbilical cord	Allogeneic	Artificial tears	UC-MSC-Exos 10ug/drop, four times a day for 14 days
8	NCT04388982	the Safety and the Efficacy Evaluation of Allogeneic Adipose MSC-Exos in Patients With Alzheimer’s Disease	1/2	9	Alzheimer’s disease	Non-randomized	Adipose tissue	Allogeneic	Nasal drip	5/10/20 μg ASC-Exos Twice a week Duration: 12 weeks
9	NCT04173650	MSC-EVs in Dystrophic Epidermolysis Bullosa	N/A	10	Dystrophic epidermolysis bullosa	Single group assignment	Bone marrow	Allogeneic	Local	N/A
10	NCT04602104	A Clinical Study of Mesenchymal Stem Cell Exosomes Nebulizer for the Treatment of ARDS	1/2	169	ARDS	Randomized, double-blinded, controlled	N/A	Allogeneic	Inhalation	
										Low: 2.0 × 10^8^ exosomes;
										Medium: 8.0 × 10^8^ vesicles;
										High: 16.0 × 10^8^ exosomes
										(once daily for a week)
11	NCT04544215	A Clinical Study of Mesenchymal Progenitor Cell Exosomes Nebulizer for the Treatment of Pulmonary Infection	1/2	60	Pulmonary infection	Randomized, double-blinded, controlled	Adipose tissue	Allogeneic	Inhalation	Low: 8.0 × 10^8^ exosomes/3 ml;
										High: 16.0 × 10^8^ exosomes/3 ml
										(once daily for a week)
12	NCT04270006	Evaluation of Adipose Derived Stem Cells Exo.in Treatment of Periodontitis (exosomes)	1	10	Periodontitis	Open label	Adipose tissue	Autologous	Local	N/A
13	NCT03608631	iExosomes in Treating Participants with Metastatic Pancreas Cancer With KrasG12D Mutation	1	28	Metastatic pancreas cancer	Open label	N/A	Allogeneic	IV	N/A
14	NCT05060107	Intra-articular Injection of MSC-derived Exosomes in Knee Osteoarthritis	1	10	Osteoarthritis	Open label	N/A	Allogeneic	Intra-articular	Single dose (3–5 × 10e11 particles/dose)

*Abbreviations* BM-MSC-Exos: Bone marrow mesenchymal stem cell-derived exosomes, ASC-Exos: Adipose tissue mesenchymal stem cell-derived exosomes, UC-MSC-Exos: umbilical cord mesenchymal stem cell-derived exosomes. UCB-MSC-Exos: umbilical cord blood mesenchymal stem cell-derived exosomes. GvHD: Graft-versus-host disease

Data are compiled from https://clinicaltrials.gov/

## Sources, doses, and administration route of MSC-Exos

Though MSCs can be isolated from many tissues, currently only MSCs isolated from the adipose tissue, bone marrow, or umbilical cord are used as sources of exosomes in 21 registered clinical trials. While bone marrow is the most popular source of MSC-Exos in preclinical studies [41], adipose tissue is the most frequently used source in clinical studies, as they have been used in 7 studies, followed by the bone marrow in 5 studies and umbilical cords in 4 studies. All except two studies used allogeneic sources due to the non-immunogenic effect of MSC-Exos.

Intravenous infusion, inhalation, or local administration are routes of administering MSC-Exos in clinical studies. Specifically, 6 studies used IV infusion or inhalation, while 5 studies employed a local administration of MSC-Exos. The dose of administration in these clinical studies varied depending on the route of delivery and the disease. Furthermore, there were variations in the units with which MSC-Exos were calculated; i.e., some studies calculated the amount of MSC-Exos by their weight in micrograms, some by the number of particles, while others simply stated the number of MSCs used to generate MSC-Exos (Table 1). Therefore, there is currently no consensus on the dose of MSC-Exos used, and it is therefore challenging to compare the doses among different studies.

## Methods of MSC-Exos isolation

MSC-Exos to be used in clinical trials must be produced under the GMP-grade condition in which the cell culture environment, cultivation system, dissipation enzyme, and culture medium are strictly monitored using GMP standards. MSC-Exos need to be purified and characterized for physical structure and bioactivity function and later pass the product release criteria before being used in clinical trials. The ultracentrifugation method is the most frequently used for isolating MSC-Exos in clinical trials. Suspension components are separated using centrifugation based on their sizes, shapes, densities, centrifugal vigor, and solvent stickiness. Significant centrifugal forces at up to 1,000,000*g* were utilized in ultracentrifugation to separate MSC-Exos from various sample components [43]. The tangential flow filtration system (TFF) is another method to concentrate condition medium and purification of MSC-Exos based on vesicle sizes. When using this method, a cell culture medium is filtered with a sterile hollow fiber polyether–sulfone membrane with a specific pore size (in µm) to remove the cell debris and retain the biomolecules. Next, the filter is washed with sterile phosphate-buffered saline (PBS) several times. After washing, the MSC-Exos is concentrated and diafiltrated using a sucrose buffer [44]. All the published clinical trials on MSC-Exos used the ultracentrifugation method except Cho et al. [45] and Kwon et al. [46], who used the TFF method during MSC-Exos preparation.

## Characterization of MSC-Exos

MSC-Exos intended to be used in clinical trials should meet the minimal characterization criteria for extracellular vesicles as stated in the MISEV2018 guidelines which include both marker and physical characterizations. Marker characterization should be evaluated by (i) positive for at least three protein markers of EVs, including at least one -transmembrane/lipid-bound protein -cytosolic protein and (ii) negative for at least one protein marker. Physical characterization should be evaluated by two different but complementary techniques such as electron microscopy and single particle analyzers to evaluate the size and concentration of MSC-Exos [47].

**Fig. 2:**
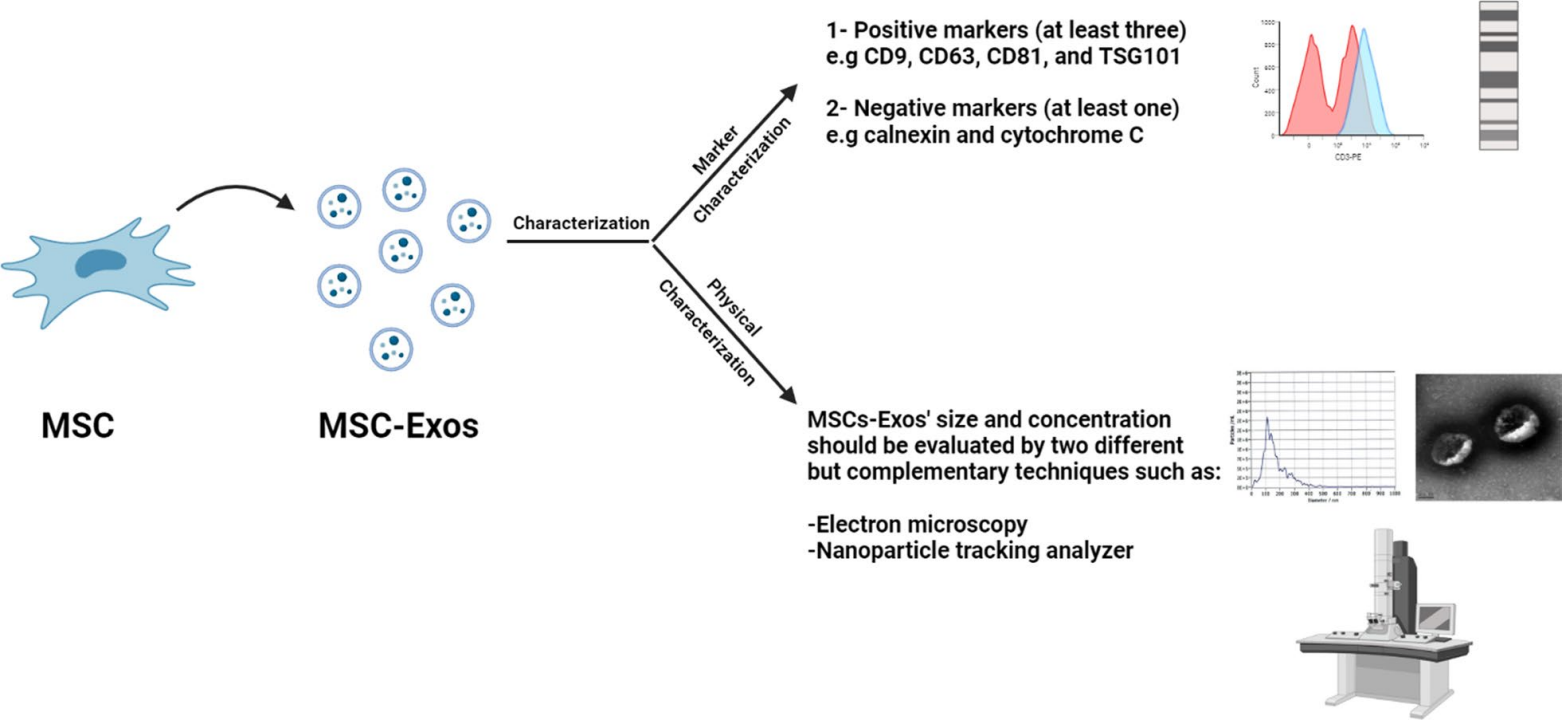
Summary of the characterization steps for MSC-Exos

Many studies have found that all MSC-Exos share markers CD9, CD63, CD81, and TSG101 [48, 49] and do not express calnexin and cytochrome C [50]. Therefore, most published studies on MSC-Exos used these markers for MSC-Exos characterization [51] as well as electron microscopy and nanoparticle tracking analyzer to evaluate physical characteristics (Fig. 2).

### Published clinical studies using MSC-Exos therapy

#### MSC-Exos for chronic kidney disease

Chronic kidney disease (CKD) is a progressive and irreversible disease that happens after the decline of renal functions to a specific threshold [52]. Progressive tubulointerstitial fibrosis is a common characteristic of CKD leading to end-stage renal disease (ESRD) [53]. The first reported clinical trial using MSC-Exos in CKD was a single-center, randomized, placebo-controlled clinical study that used umbilical cord blood MSC-Exos (UCB-MSC-Exos) to ameliorate disease progression done in Egypt by Nassar et al. [54]. The study enrolled 40 patients, aged 26 to 44 years, with stage III or IV CKD; Participants were randomized into control (placebo) or UCB-MSC-Exos groups at a 1:1 ratio. Those assigned to the UCB-MSC-Exos group received a weekly dose of Exos at 100 μg/kg body weight for two weeks. The first dose was administered intravenously and the second intra-arterially. The control group received intravenous saline infusion. The UCB-MSC-Exos group achieved a significant improvement in estimated glomerular filtration rate (eGFR), serum creatinine level, blood urea, and urine albumin-to-creatinine ratio (UACR) during the 12-month study period. The improvements were likely due to an increase in circulating anti-inflammatory cytokines and a decline in proinflammatory cytokines, i.e., plasma TGF-β1 and IL-10 levels were significantly higher in the treated group than the control group. Additionally, the treated group exhibited a substantial decline in plasma TNF-α levels compared with the controls. Moreover, kidney biopsies from the treated group only showed mRNA expression of CD133 and Ki67 in tubular epithelial cells, indicating the growth and/or dedifferentiation of the tubular cells after UCB-MSC-Exos infusion [54].

#### MSC-Exos for skin hyperpigmentation

Hyperpigmentation of the skin is a dermatological disorder that affects the skin color by making it discolored or darkened [55]. Current treatment strategies take a long time to cure the disease, and the results were not ideal. Additionally, these treatment strategies have many limitations and side effects including erythema, skin peeling, and drying [55]. A prospective, split-face, randomized placebo-controlled study investigated the therapeutic effect of exosomes derived from adipose tissue-derived MSCs (ASC-Exos) on skin hyperpigmentation. Twenty-one females aged between 39 and 55 years with hyperpigmentation participated in the study. The treated group received 0.2 g of ASC-Exos (size range 30–200 nm) in a cosmetic formulation, and the control group received 0.2 g of placebo (cosmetic formulation without ASC-Exos) twice per day for 8 weeks. The ASCA-Exos significantly reduced the amount of melanin in the treatment group compared to the control group; however, the improvement only lasted for 2 months and the disease condition relapsed over time [50]. A potential mechanism was that ASC-Exos induced synthesis of ceramide or sphingosine 1-phosphate that controls melanogenesis in melanocytes. Another potential contributor is that ASC-Exos constitute microRNAs such as has-miR-137 has-miR-145 and has-miR-330 that have been shown to reduce melanin levels in melanoma cells [56–58].

#### MSC-Exos for acne scars

Facial atrophic acne scarring is a disfigurement of the face that may cause social isolation and other issues. One of the common treatment methods for this condition is fractional carbon dioxide laser (FCL) resurfacing [59, 60]. Although FCL is efficacious in treating acne scars, better scar reduction with less severe adverse effects during post-procedural wound healing is still needed [46].

In a study done in Korea, 25 participants aged between 19 and 54 years (18 males and 7 females) volunteered to test the effect of ASC-Exos in combination with FCL in facial acinar scar healing. Among them, 12 with Fitzpatrick skin type III and 13 with type IV were tested in two separate stages for 12 weeks. In the first treatment group, the entire face of each patient using 10,600-nm FCL was followed by washing the face with mild soap topical anesthesia utilized by EMLA® cream for 30 min before the laser therapy. After the laser treatment, the participants were randomly assigned to receive 1 ml of ASC-Exos or control gel to put on their entire faces. In the next two days, the participants applied each solution to a specified side of their faces twice per day. The side treated with ASC-Exos achieved remarkably higher enhancement than the control sides. The treatment-related erythema was milder on the sides treated with ASC-Exos. The results demonstrated that the size of skin pores and skin surface scabrously declined from baseline on the ASC-Exos side, while there were no observable changes on the control side. Thus, combining ASC-Exos and laser therapy could provide synergistic influences on the efficiency of atrophic acne scar treatments [61]. Potential mechanisms of improvement include exosomes supplying multiple anti-inflammatory and regenerative growth factors, optimizing characteristics of fibroblast accelerating wound healing and wound repair, and increasing de novo synthesis of ceramides [61].

#### MSC-Exos for graft-versus-host disease (GvHD)

Allogeneic hematopoietic stem cell transplantation (HSCT) is a potential life-saving procedure to treat patients with hematologic malignancies. One of the most severe complications associated with HSCT is acute or chronic graft-versus-host disease (GvHD), a complex immunologic process that occurs on a pathobiological spectrum. GvHD is a leading cause of long-term morbidity and impaired health-related quality of life [62]. The current treatment for GvHD is broad systemic immunosuppression agents that have low response rates and increased risk for opportunistic infection [63].

According to a case report, exosomes derived from the supernatants of BM-MSC were administered to a patient with GvHD. The dose of the exosomes was 1 unit (exosomes extracted from a conditioned medium of 4 × 10^7^ BM-MSCs) per day for two days. The dosage was then increased incrementally and administered every 2–3 days until it reached 4 units per day. To monitor the possible effect of MSC-Exos on the immune response of the patient’s PBMCs, blood samples were taken after each administration. Pro/inflammatory cytokines such as IL-1β, TNF-α, and IFN-ɣ were evaluated and the data showed a reduction in pro/inflammatory cytokines secreted by patient’s PBMCs. There was a dramatic improvement in the symptoms; the diarrhea quantity was significantly decreased, and cutaneous and mucosal GvHD showed a notable restrain within 14 days. However, the patient died from pneumonia 7 months after therapy. Nevertheless, the results were promising and showed potential efficiency to cure GvHD [64]. It was also speculated that MSC-Exos impaired the in vivo capability of the patients’ PBMC to release proinflammatory cytokines that contributed to their therapeutic effects [64].

#### MSC-Exos for COVID-19

COVID-19 is a respiratory disease caused by SARS-CoV-2, a coronavirus discovered in 2019. The infection occurs due to the presence of the genetic material, RNA, of the *genus Betacoronavirus* of the Coronaviridae family. Extreme and uncontrolled responses of the immune system besides fatal cytokine storms play a critical role in the pathogenesis of acute respiratory distress syndrome (ARDS) caused by SARS-CoV-2 infection. Major components of ARDS pathogenesis include the breakdown of cytotoxic mechanisms, over-activation of cytotoxic lymphocytes and macrophages with the excessive release of proinflammatory cytokines (IL-1, IL-2, IL-6, IL-8, IL-10, granulocyte colony-stimulating factor, and monocytic chemoattractant protein 1, MCP-1), inflammatory markers (CRP, serum ferritin), and infiltration of organs and tissues by activated T-lymphocytes and macrophages, causing hyperinflammatory reactions. These acute lesions can result in lung damage and rehabilitation after discharge or death. Exosomes can orchestrate inflammatory and regenerative processes due to the alteration in the concentration of anti-inflammatory cytokines and the immune cell transformation to regenerative secretome. Exosome inhalation is thought to decrease inflammation and lung damage, inducing regenerative processes, and has a potential therapeutic effect in the treatment of COVID-19 [65].

A prospective open-label cohort clinical study has been conducted to test the safety and efficacy of MSC-Exos as a therapy for severe COVID-19. Exosomes derived from BM-MSCs (product named: ExoFlo) were used. 24 participants received an intravenous dose of ExoFlo which contain exosomes produced from 1–10 × 10^6^ MSCs/kg, and the treatment safety was assessed daily for two weeks after administration. The participant survival rate was 83%; about 71% of the patients were cured, 13% remained in critical but stable condition, and 16% died for reasons unrelated to the treatment. Generally, after a single dose, the clinical status of the participants and oxygenation was increased with an average pressure of arterial oxygen to fraction of inspired oxygen ratio (PaO2/FiO2) increasing by approximately 192%. Furthermore, the therapy reduced the C-reactive protein ratio by 77%, ferritin by 43%, and D-dimer by 42%. The significant improvement of lymphopenia as indicated by increased CD3^+^, CD4^+^, and CD8^+^ T cells after MSC-Exos injection suggests that the therapeutic mechanism of action of MSC-Exos was due to their immunomodulation effects. Thus, the authors suggested that MSC-Exos are a promising treatment for COVID-19 [66].

#### MSC-Exos for osteoarthritis

Osteoarthritis (OA) is a type of arthritis that affects the joints, causing pain and stiffness. It is the most common form of arthritis and typically affects the joints in the hands, hips, knees, and spine. There is no cure for OA, but to reduce the pain by using disease-modifying drugs such as NSAIDs, acetaminophen, and opioid analgesics [67].

In this study, BM-MSC-Exos have been tested as curative agents on the osteoarthritis of different joints, including the knee, shoulder, elbow, hip, ankle, and wrist. Participants included 33 Navy SEAL veterans, who were injected with a 2-ml single dose of ExoFlo, a BM-MSC-Exos product. The joints in the study were divided in knees (*n* = 58), shoulders (*n* = 32), elbows (*n* = 16), hips (*n* = 12), ankles (*n* = 8), and wrists (*n* = 6).

Brief Pain Inventory and Oswestry Disability Index scores were significantly improved by 77% and 80%, respectively, at 6 months post-infusion. Moreover, the Upper Extremity Functional Scale and Lower Extremity Functional Scale were improved by 51% and 76%, respectively. The adverse effects reported for 24 h included backache in one patient, the elevation of pain in the injected joint in 4 participants, and alterations in gut habits in one participant. In addition, one participant suffered from sleep disruptions for 2 nights. These results suggest that treatment of OA joints using BM-MSC-Exos is efficacious and safe and may subrogate joint replacement surgery [68].

#### Clinical safety of MSC-Exos on healthy volunteers

In a phase 1 single-arm clinical trial, Shi et al. (2021) examined the safety of nebulized allogeneic ASC-Exos in 24 healthy volunteers who received 2–16 × 10^8^ particles by inhalation. The ASC-Exos were characterized by CD9, CD63, CD81, and TSG101 expression and ASC-Exos size and concentration were evaluated using nanoparticle tracking analysis. For the follow-up and safety monitoring, several biological tests, such as blood parameters, liver and kidney function, lactate dehydrogenase, immunoglobulin concentration, and cardiograms, were performed one week after infusion. All the volunteers tolerated the infusion well and did not show adverse reactions within the week after nebulization [69].

### Ongoing MSC-Exos clinical studies

#### MSC-Exos in stroke

Stroke is ranked as the second leading cause of death and represents a heavy burden to society globally [70]. Acute ischemic stroke (AIS) accounts for more than 70% of strokes [71]. AIS is characterized by the unexpected loss of blood circulation to the brain leading to loss of neurologic function. It is caused by thrombotic or embolic obstruction of a cerebral artery [72]. An ongoing clinical trial led by Zali et al. (www.clinicaltrials.gov, NCT03384433) evaluates the efficacy of BM-MSC-Exos transfected by miR-124 in patients diagnosed with acute ischemic stroke. In this phase 1/2 trial, 5 participants, aged between 40 and 80 years, received one 200-mg dose of total protein BM-MSC-Exos transfected with miR-124 in the ischemic area using stereotactic guidance one month after the stroke. The safety of the participants will be followed for 12 months after the therapy and used as the primary endpoints. Parameters to be measured include the registration of side effects such as recurrent stroke, brain edema, seizures, and ischemic to hemorrhagic transformation. Moreover, the potential efficacy will be measured by the amelioration in the manipulated Rankin Scale during the first 12 months post-treatment, and through measuring the degree of AIS patients’ disability using a score from 0 to 6.

#### MSC-Exos in Alzheimer’s disease

Alzheimer’s disease (AD) is one of the most common neurodegenerative diseases worldwide. It is known to cause loss of memory, cognitive impairment, changes in behavior, and loss of functional abilities [73]. The pathological hallmark of AD is the deposition of amyloid β plaques and intracellular neurofibrillary tangles (NFT) that hinder the trafficking of many nutrients to the brain [74]. The abnormal aggregation of intracellular Tau protein to constitute NFT, neuritic plaques, and neuron death [75] are major features of AD. Most of the available drugs for AD are only effective for those with moderate symptoms with no effect in preventing neural loss or progressive deterioration of cognition [76]. In an ongoing phase 1/2 clinical trial led by Wang and colleagues (NCT04388982), allogeneic ASC-Exos are given to 9 participants aged 50 or older with Alzheimer’s disease. The major goal of this trial is to test the efficacy of MSC-Exos in the treatment of mild to moderate dementia caused by AD. The participants in this study are divided into 3 groups. The first group is administered a low dose of ASC-Exos at 5 μg via nasal drip twice per week for 12 weeks. The second group received a medium dose of ASC-Exos at 10 μg via nasal drip twice per week for 12 weeks. Lastly, the third group receives a high dose of ASC-Exos at 20 μg for 12 weeks. The primary endpoint will be measured by the number of treated patients who show abnormal values for both liver and kidney functions and those who show adverse events within a time frame of 3 months. Additionally, their cognitive scale and functional ability will be evaluated via the cognitive subscale (ADAS-cog) and ADCS-ADL scores, respectively, within different time frames.

#### MSC-Exos for Type 1 diabetes mellitus (T1DM)

T1DM is an autoimmune disease that is caused by the destruction of insulin-secreting pancreatic β-cells that leads to the elevation in glucose levels in the blood (hyperglycemia) [77]. Currently, there is no cure for T1DM, and treatment with insulin injections is the only option [78]. In a study led by Nassar et al. at the Sahel Teaching Hospital and Cairo University (NCT02138331), UCB-MSC-Exos are tested in T1DM patients aged between 18 and 60 years with reduction of C-peptide chain more than 50%, C-peptide of more than 0.8 ng/mL at Screening, and requiring insulin ≥ 0.4 IU per kg per day.

Twenty patients will receive two successive doses intravenously. The first dose will be purified exosomes, 40–180 nm, extracted from the supernatant generated from 1.22–1.51 × 10^6^ MSCs/kg. The second dose containing MVs with the particle size range of 100–1000 nm, will be administered to patients 7 days after the first dose. The exosomes will be distinguished by the presence of markers CD63, CD9, Alix, TSG101, and HSP 70, while the MVs will be characterized by markers annexin V, Flotilin-2, selectin, integrin, and CD40 metalloproteinase. The duration of the study is 3 months. Liver and kidney functions, HbA1c, glucose tolerance, fasting, and 2-h postprandial blood glucose levels, C-peptide chain levels, and insulin doses will be measured during the study. The primary endpoint will be the change of dosage of the insulin of the treated T1DM patients within a 3-month time frame. Additionally, β-cell mass and HbA1c levels will be measured at the end of the study and compared to the baseline.

#### MSC-Exos for pancreatic cancer (PC)

PC is known to be the fiercest cancer among all known cancers with a mortality rate of around 83% [79]. The median survival rate of PC patients is 4.1 months with an overall survival rate of less than 5% [80]. At the time of diagnosis, more than 85% of PC patients have metastatic disease. Most surgical or medical interventions are not effective for PC. Additionally, the limited efficacy of the treatments contributes to high mortality rates. Scientists have revealed that there is a genetic lesion related to PC [81]. Mutation of the Kirsten rat sarcoma virus (Kras) gene was found in 75 to 90% of the cases which represents the early event in the development of the PC malignancy [82].

A Phase 1 study in MD Anderson Cancer Center (NCT03608631) evaluates the suitable dose and adverse events of MSC-Exos with Kras^G12D^ siRNA for patients with metastatic pancreatic cancer and the KrasG12D mutation. Twenty-eight adult patients aged 18 or older, will receive the MSC-Exos intravenously. Participants will be given MSC-Exos over 15–20 min on days 1, 4, and 10, and the administration will be repeated every 14 days for up to 3 courses. The primary objectives of this study are to define both the maximum tolerated dose (MTD) and the dose-limiting toxicities (DLT) of MSC-Exos with KRAS^G12D^ siRNA for PC patients.

#### MSC-Exos for macular degeneration

A macular hole is a retinal tissue disorder involving the anatomic fovea which in turn affects central visual acuity. It has been linked to many ocular conditions and was originally described in the settings of trauma [83]. The cause of macular holes is still unknown; however, one study has stated that tangential vitreous traction may be responsible for macular holes [84]. Preclinical studies have demonstrated that systematic inoculation of MSC-Exos alleviates inflammation and damage in macular degeneration [85]. An ongoing clinical trial led by Zhang et al. (NCT03437759) will assess the safety and efficacy of MSCs and MSC-Exos for promoting the healing of large and refractory macular holes. In this trial, 44 participants, including children and adults up to 80 years old, who were diagnosed with early phase 1 macular holes will receive one 50 or a 20-μg dose of MSC-Exos via local injection. The participants will be followed up for 6 months with best-corrected visual acuity measurement, fundoscopy, optical coherence tomography, and physical examination [86]. The results of this study will be assessed by measuring the minimum linear diameter of the hole measured by spectral-domain optical coherence tomography (OCT) 24 weeks after the surgery.

#### MSC-Exos for COVID-19

In the study led by the Clinics of the Federal State Budgetary Educational Institution SSMU and Samara Regional Clinical Hospital V.D. Seredavin (NCT04491240), 30 patients aged between 18 and 65 years, and confirmed for COVID-19 by PCR analysis, will be randomized into 3 groups. Two groups, phase 1 and 2, are treated with exosomes, while the third group is the control. Those assigned to the treatment groups will be given 3 ml of a special solution containing two different types of 0.5–2 × 10^10^ nanoparticles twice a day for 10 days via inhalation. Those assigned to the control group will receive the same volume of the special solution without nanoparticles. The primary endpoint will be the number of participants with non-serious and serious adverse events within 30 days after clinic discharge, and the number of participants with non-serious and serious adverse events during the Inhalation Procedure (after each inhalation within 10 days).

Shanghai Public Health Clinical Center and Wuhan Jinyintan Hospital in Wuhan, China (NCT04276987) lead another trial using ASC-Exos as a treatment for COVID-19. In this trial, 24 participants diagnosed with COVID-19 will be given 5 aerosol inhalation doses of ASC-Exos at 2.0 × 10^8^ nanovesicles/3 ml for the first 5 days. The safety and efficacy profiles, including the frequency of adverse event (AE) and severe adverse event (SAE), will be evaluated 28 days after the first dose. The primary outcomes will be (i) AE and SAE (time frame: up to 28 days) and time to clinical improvement (time frame: up to 28 days).

#### MSC-Exos for pulmonary infection

Pulmonary infection is a critical disease threatening human health. As a consequence of increased drug resistance caused by extended use of antibiotics, the mortality rate after pulmonary infection is elevated due to the lack of effective therapies and poor diagnosis. Treatment with glucocorticoids and immunomodulators is not effective and may lead to heavier use of antibiotics.

In a clinical study (NCT04544215), an aerosol inhalation of allogeneic ASC-Exos will be utilized to treat pulmonary infections by Gram-negative *Bacilli* resistant to carbapenems. Sixty participants between the age of 18 and 75 will be randomly divided into low-dosage, high-dosage, or placebo groups. The low-dosage groups will receive 7 doses aerosol inhalations of 8.0 × 10^8^ nanovesicles in 3 ml volume and the high-dosage group will receive 7 doses of aerosol inhalation of 16.0 × 10^8^ nanovesicles in 3 ml of volume.

#### MSC-Exos for acute respiratory distress syndromes (ARDS)

ARDS is a life-threatening condition where the lungs cannot supply sufficient oxygen to the body’s vital organs [87]. As estimated by the 2012 Berlin diagnostic criteria, of all patients admitted to the ICU, approximately 10% have ARDS. Recently, elevated levels of ARDS have increased the social and economic burden. Although there are basic therapies including ventilation techniques to enhance hypoxia and fluid management, there is a lack of robust medication measurements [87]. According to several studies, MSC-Exos can boost different pathological modifications resulting from a lung infection, such as decreased pulmonary edema, reduce alveolar inflammation, and bacterial infections, suggesting a potential use in the treatment of ARDS [65].

In a clinical trial targeting ARDS (NCT04602104), researchers aim to assess the efficacy of an aerosol inhalation of allogeneic MSC-Exos for ARDS. In this study, 169 participants, ages between 18 and 70 years, will be randomized into two phases with 3 groups in each one. The three groups of the phase 1 study included a low-dose group receiving an aerosol inhalation of 2.0 × 10^8^ MSC-Exos particles once daily for a week, a medium-dose group receiving an aerosol inhalation of 8.0 × 10^8^ MSC-Exos particles once daily for a week, and the high-dose group receiving an aerosol inhalation of 16.0 × 10^8^ MSC-Exos particles once daily for a week. During phase 2, the protocol includes basic treatment and MSC-Exos inhalation. The first group will be administered the basic treatment and an aerosol inhalation of MSC-Exos (a quarter of MTD) once daily for a week, while the second and third groups will receive an aerosol inhalation of MTD and normal saline once daily for a week.

#### MSC-Exos for multiple organ dysfunction syndrome (MODS) after surgical repair of acute Type A aortic dissection

MODS is a common debilitating condition after surgical repair for acute type A aortic dissection (ATAAD) [88]. MODS is one of the primary reasons of postoperative death and is responsible for about half of the postoperative deaths that occur after ATAAD [88]. MODS is a systemic, dysfunctional inflammatory response that requires a long ICU stay. Despite the modern advancements in surgeries, the death rate continues to rise for these critical care conditions. In vivo studies have revealed the beneficial effect of MSC-Exos in ischemia–reperfusion injuries of the heart, lung, kidney, brain, and liver [89].

A study registered by Chen and colleagues at Fujian Medical University (NCT04356300) will test the role of MSC-Exos in the prevention and treatment of MODS after ATAAD. This randomized study has two parts. In the first part (prevention scheme): 15 patients will be administered 150 mg of MSC-Exos intravenously once a day for 14 days immediately after ascending aortic replacement, and the other 15 patients (control) will be treated with a basic standard of care (SOC). In the second part (treatment scheme): 15 patients will be administered the same dose of MSC-Exos for the same number of treatments but after the onset of MODS and ascending aortic replacement combined with the open placement of triple-branched stent graft for ATAAD, and the other 15 patients receive SOC and as the control. The short and long-term side effects of MSC-Exos include anaphylactic reactions and oncogenicity, and a sequential organ failure assessment score will be evaluated for up to 6 months. Furthermore, the therapeutic impacts on the enhancement of liver, lung, and coagulation function will be assessed by bilirubin levels, oxygenation index, and blood platelet counts, respectively.

#### MSC-Exos for dystrophic epidermolysis bullosa

Dystrophic epidermolysis bullosa (EB) is a rare inherited blistering disease caused by mutations of the collagen type VII alpha 1 (COL7A1) gene. The dysfunction of type VII collagen causes subepidermal blistering below the lamina densa, leading to conditions such as intractable ulcers, wide-ranging scarring, malnutrition, and malignancy [90]. In a phase 1/2 single-assignment open-labeled clinical trial, the safety and efficacy of BM-MSC-Exos (Product name: AGLE-102) are tested on the lesions of 10 patients with EB. This study, which will be performed by Aegle Therapeutics (NCT04173650), includes 10 participants with only a single wound, aged 6 years or older, who will be given 6 doses of BM-MSC-Exos over a 3-month period. The primary endpoint will be dose-limiting toxicity which examines a dose escalation and dose-limiting toxicity defined as in the NCI/CTCAE v4.0 grading scale). The secondary endpoint will be wound size evaluation.

#### MSC-Exos for dry eye in patients with chronic GVHD

Among patients with chronic GvHD (cGVHD), 60–90% of them are diagnosed with dry eye symptoms followed by dryness, foreign body sensation, and photophobia, which may cause blindness. Artificial tears, lacrimal punctum embolization, and local immunosuppressants are used to treat the dry eyes symptoms associated with cGVHD. However, the overall effectiveness is still inadequate; there are some side effects and the course of treatment is lengthy and expensive. Therefore, clinicians must investigate novel approaches to treating dry eyes associated with cGVHD to increase patient survival and quality of life. Meanwhile, UC-MSC-derived exosomes (UC-MSC-Exos) were shown to decrease dry eye symptoms in vivo in preclinical studies [91]. The clinical study, which will be conducted by Zhongshan Ophthalmic Center, Sun Yat-sen University (NCT04213248), aims to determine whether UC-MSC-Exos could alleviate dry eye symptoms in patients with cGVHD. In this open-label single-assignment study, the effect of UC-MSC-Exos will be tested on 27 participants between the ages of 18 to 70 years. Participants will be administered with artificial tears for 2 weeks followed by UC-MSC-Exos at 10 ug/drop 4 times a day for 2 weeks. Participants will be followed up for 12 weeks to measure the dry eye progression. The primary endpoint will be a change in Ocular Surface Disease Index (OSDI) score. Secondary endpoints include tear secretion, tear break time, areas stained by fluorescent, ocular redness, tear meniscus, and best-corrected visual acuity, to be measured at 3 days, 2, 3, 4, 6, 8, 10, and 12 weeks post-treatment.

#### MSC-Exos for periodontitis

Periodontitis is an infection-driven inflammatory non-communicable disease that impacts the periodontium resulting in unrepairable damage. The disorder may only show little or moderate symptoms over many years [92]. In an ongoing phase 1 open-label clinical trial (NCT04270006), a team from Beni-Suef University and Cairo University in Egypt plan to evaluate the therapeutic effect of autologous ASC-Exos on scaling and root planning in the treatment of periodontitis. This will be a single-assignment intervention that will enroll 10 participants between the ages of 18 and 50 years. Participants will receive MSC-Exos locally into the periodontal pockets and followed for 6 months after treatment. Primary outcomes will include change in gingival inflammation, pocket depth, attachment level, and bone level by cone beam computed tomography at 0, 3, and 6 months post-treatment.

### Future perspective of MSC-Exos therapy

In the past several years, MSC-Exos therapy has become a novel option for the treatment of many diseases [12]. The number of industrial companies interested in MSC-Exos as a therapeutic tool has risen to about 45 worldwide [93]. Furthermore, the innate capacity of MSC-Exos to transfer genetic materials, protect them from degradation by various factors, and deliver these genetic materials to recipient cells at high selectivity indicates that MSC-Exos is an exemplary delivery system for small particles. Thus, they may be effective in facilitating gene therapies for cancer and treatments of other debilitating disorders and potentially used in regenerative medicine [94]. Additionally, several preclinical studies have reported that MSC-Exos therapy is superior compared to cell therapy in terms of safety, efficacy, and versatility [95, 96]. However, there is a need to examine and address the adverse effects of MSC-Exos therapies to broaden their clinical utilization [97]. Thus, more preclinical studies are needed to optimize the suitable doses, route of administration, and sources of MSCs.

There are also some challenges in using MSC-Exos in the clinic. For instance, the fast clearance of MSC-Exos from the body might limit their long-term therapeutic effects [98]. Also, the heterogeneity of MSC-Exos due to different culture conditions and cell passages is another challenges. To overcome these obstacles, standardization of MSC-Exos isolation protocols is required. By using consistent methods for isolating exosomes, researchers can minimize variations on exosome size, composition, and function. Moreover, using defined culture conditions can help to produce more homogenous populations of MSCs and their exosomes. Furthermore, generating well-tested immortalized MSC lines could be a solution to generate homogenous MSC-Exos. Another challenge is the long-term preservation to extend the shelf life of MSC-Exos. Several research groups are working on lyophilizing MSC-Exos or by adding stabilizing agents such as sugars or polyethylene glycol to help to preserve exosomes for longer periods of time [99, 100]. Moreover, current efforts also focus on using encapsulation to enhance their persistence in the body [101]. For example, with the advancements in bioengineering and cellular manipulation technologies, the upcoming trend regarding exosome utilization will be the engineering of exosomes which will give the chance to be more specific and be used in highly complicated areas of medicine [102]. These additional studies aimed to improve the efficacy of exosomes will further enhance the potential of MSC-Exos as a novel therapeutic approach for disease treatment.

## Conclusion

MSC-Exos is becoming a novel and promising cell-free therapeutic tool in multiple ongoing clinical studies for various diseases and demonstrated safety and potential efficacy in a handful of reported clinical studies. On the other hand, MSC-Exos clinical applications also face challenges, such as product heterogeneity, fast clearance from the body, and long-term preservation stability. Moreover, there is an urgent need to standardize the therapeutic doses, route of administration, and source of parent MSCs for the MSC-Exos product. Thus, more preclinical and clinical studies are required to push the MSC-Exos therapy into clinical applications.

## Data Availability

Not applicable.

## Abbreviations

MCs: Mesenchymal stromal/stem cells (MSC)
BM-MC-Exos: Bone marrow mesenchymal stem cell-derived exosomes
ASC-Exos: Adipose tissue mesenchymal stem cell-derived exosomes
UC-MC-Exos: Umbilical cord mesenchymal stem cell-derived exosomes
UCB-MC-Exos: Umbilical cord blood mesenchymal stem cell-derived exosomes
GvHD: Graft-versus-host disease
TFF: Tangential flow filtration system
MISEV: Minimal Information for Studies of Extracellular Vesicles
CKD: Chronic kidney disease
ARDS: Acute respiratory distress syndrome
AIS: Acute ischemic stroke
AD: Alzheimer’s disease
T1DM: Type 1 diabetes mellitus
PC: Pancreatic cancer
MODS: Multiple organ dysfunction syndrome
ATAAD: Acute type A aortic dissection
